# Introduction to European comments on “Medullary Thyroid Cancer: management guidelines of the American Thyroid Association”

**DOI:** 10.1186/1756-6614-6-S1-S1

**Published:** 2013-03-14

**Authors:** Barbara Jarzab, Ulla Feldt-Rasmussen

**Affiliations:** 1 Department of Nuclear Medicine and Endocrine Oncology, Maria Sklodowska-Curie Memorial Cancer Center and Institute of Oncology, Gliwice Branch, Wybrzeze Armii Krajowej 15, 44-101 Gliwice, Poland; 2 Medical Endocrinology PE 2132 Rigshospitalet, Copenhagen University Hospital Blegdamsvej 9, DK-2100 Copenhagen, Denmark

## Abstract

Guest Editors of *Thyroid Research* supplement devoted to medullary thyroid cancer present the history on how the discussion about “Medullary Thyroid Cancer: management guidelines of the American Thyroid Association” was initiated and subsequently widely commented before and during European Thyroid Association – Cancer Research Network Meeting in Lisbon. It is explained why it has been decided to publish the manuscripts within the supplement – to document voices from the discussion and popularize them.

## 

The publication of important paper by R. Kloos et al. “Medullary Thyroid Cancer: management guidelines of the American Thyroid Association” [1] has been a great achievement in the world of thyroidology in general and thyroid cancer in particular. The guidelines were evidence based for management of medullary thyroid cancer, a very rare cancer type, known well only to few thyroidologists. These were crucial factors for their endorsement by the European Thyroid Association. However, among some European thyroidologists the document raised a vivid online discussion, which led to the organization of this topic as the program of the European Thyroid Association – Cancer Research Network Meeting (ETA-CRN) in Lisbon in September 2009. The program of this event is presented in Additional file 1. About 140 participants, mostly European thyroidologists, took part in the discussion [2]. This supplement of *Thyroid Research* is to summarize aroused debate on the subjects in question. The publication followed the request from ETA-CRN General Assembly in Pisa, 2012 and the disputants were invited to present their views. Therefore, Ulla Feldt-Rasmussen, President of ETA-CRN and Barbara Jarzab, Secretary of ETA-CRN undertook the role as Guest Editors of this supplement.

We are very thankful that the authors agreed to join the supplement on a voluntary basis and undertook the challenge to formulate conclusions from Lisbon and update them. We believe that more than a 3-year long delay allowed to balance and objectify the assessment of the ATA medullary thyroid cancer guidelines well. We have also invited colleagues, members of ETA-CRN, who took active part in the online discussion but did not have the possibility to join discussion in Lisbon [3,4]. Please note, that all invited articles have been peer reviewed before publication, according to BioMed Central Guidelines for externally administered peer-review. We asked all peer reviewers to declare their competing interests in relation to reviewed paper.

We hope this supplement, devoted to medullary thyroid cancer, will enrich our knowledge about this rare disease and its treatment, and will become a positive contribution in the study of its nature.

## Competing interests

No competing interests exist for me and my co-authors.

## Supplementary Material

Additional file 1 Table 1. Program of 9^th^ ETA-CRN Annual Meeting held in Lisbon, Portugal on 5^th^ September, 2009: European Comments on Medullary Thyroid Cancer Management Guidelines of the American Thyroid Association Click here for file

## Declarations

This article has been published as part of *Thyroid Research* Volume 6 Supplement 1, 2013: European comments on Medullary Thyroid Cancer Management Guidelines of the American Thyroid Association. The full contents of the supplement are available online at http://www.thyroidresearchjournal.com/supplements/6/S1. Publication of this supplement has been supported by the European Thyroid Association-Cancer Research Network.
