# Supplementary material for: Introduction to European comments on “Medullary Thyroid Cancer: management guidelines of the American Thyroid Association”
Source: Thyroid Res. 2013 Mar 14;6(Suppl 1):S1. doi: 10.1186/1756-6614-6-S1-S1 (PMC3599712; doi:10.1186/1756-6614-6-S1-S1)
Supplement: Additional file 1 — Table 1. Program of 9th ETA-CRN Annual Meeting held in Lisbon, Portugal on 5th September, 2009: European Comments on Medullary Thyroid Cancer Management Guidelines of the American Thyroid Association [file 1756-6614-6-S1-S1-S1.doc]

**Table 1. Program of 9^th^ ETA-CRN Annual Meeting held in Lisbon, Portugal on 5^th^** September, 2009: European Comments on Medullary Thyroid Cancer Management Guidelines of the American Thyroid Association

| Title of presentation | Speaker |
| --- | --- |
| Introduction | Barbara Jarzab and Ulla Feldt-Rasmussen |
| Part 1: Issues related to diagnosis, treatment and monitoring of clinically detectable MTC | |
| 1. Calcitonin estimation in patients with nodular goiter and its significance for early detection of MTC | Rosella Elisei (Pisa, Italy) |
| 2. Serum calcitonin estimation in medullary thyroid cancer patients: basal or stimulated levels? | Chantal Daumerie (Brussels, Belgium) |
| 3. Preoperative evaluation and staging of MTC diagnosed / supposed by FNA or serum CT – role of imaging modalities (CT/MR/PET) and serum CT level. | Bruno Niederle (Vienna, Austria) |
| 4. Extent of surgery in clinically evident but operable MTC – when is central and/or lateral lymphadenectomy indicated | Sophie Leboulleux (Paris, France) |
| 5. Postoperative follow up in patients showing no evident residual disease - cut-offs for imaging/ intervention | Michele Minuto (Pisa, Italy) |
| 6. Treatment of advanced medullary thyroid cancer | Lars Bastholt (Odense, Denmark) |
| 7. Functional PET imaging and Peptide Receptor Radioisotope Therapy in MTC | Marcus Luster (Ulm, Germany) |
| 8. Clinical comments related to MTC diagnosis and management | Leonidas Duntas (Athens, Greece) |

| Part 2: Issues related to early diagnosis and management of hereditary MTC and MEN2 | |
| --- | --- |
| 9. What is the optimal range of RET mutations to be tested? | Laura Fugazzola (Milan, Italy) |
| 10. Genotype – phenotype correlations in RET mutation carriers and the ATA risk-based classification of RET mutations | Friedhelm Raue (Heidelberg, Germany) |
| 11. Timing and criteria for prophylactic thyroidectomy in asymptomatic RET carriers – the role of calcitonin serum level | Barbara Jarzab (Gliwice, Poland) |
| 12. Extent and timing of thyroidectomy in RET mutation carriers | Bruno Niederle (Vienna, Austria) |
| 13. Hyperparathyroidism in MEN2A | Maria Alevizaki (Athens, Greece) |
| 14. Diagnosis and treatment of pheochromocytoma in MEN2A | Hartmut Neumann (Freiburg, Germany) |
| Followed by: |  |
| 15. Presentation of points of general discussion and voting among the speakers | Barbara Jarzab (Gliwice, Poland) |
| 16. Results of voting among the audience | Ulla Feldt-Rasmussen and Folke Soderstrom (Copenhagen, Denmark) |
